# Microbiome-derived carnitine mimics as previously unknown mediators of gut-brain axis communication

**DOI:** 10.1126/sciadv.aax6328

**Published:** 2020-03-11

**Authors:** Heather Hulme, Lynsey M. Meikle, Nicole Strittmatter, Justin J. J. van der Hooft, John Swales, Ryan A. Bragg, Victor H. Villar, Michael J. Ormsby, Stephanie Barnes, Sheila L. Brown, Alex Dexter, Maya T. Kamat, Jasper C. Komen, Daniel Walker, Simon Milling, Emily K. Osterweil, Andrew S. MacDonald, Chris J. Schofield, Saverio Tardito, Josephine Bunch, Gillian Douce, Julia M. Edgar, RuAngelie Edrada-Ebel, Richard J. A. Goodwin, Richard Burchmore, Daniel M. Wall

**Affiliations:** 1 Institute of Infection, Immunity and Inflammation, College of Medical, Veterinary and Life Sciences, University of Glasgow, Glasgow G12 8QQ, UK.; 2 Imaging and data Analytics, Clinical Pharmacology and Safety Sciences, R&D, AstraZeneca, Cambridge CB4 0WG, UK.; 3 Bioinformatics Group, Wageningen University, Wageningen 6708 PB, Netherlands.; 4 Pharmaceutical Sciences, BioPharmaceuticals R&D, AstraZeneca, Cambridge CB4 0WG, UK.; 5 Cancer Research UK Beatson Institute, Garscube Estate, Switchback Road, Glasgow G61 1BD, UK.; 6 Centre for Discovery Brain Sciences, Simons Initiative for the Developing Brain, and The Patrick Wild Centre, University of Edinburgh, Hugh Robson Building, George Square, Edinburgh EH8 9XD, UK.; 7 Lydia Becker Institute of Immunology and Inflammation, Faculty of Biology, Medicine and Health, Manchester Academic Health Science Centre, University of Manchester, Manchester M13 9NT, UK.; 8 National Physical Laboratory, Teddington, Middlesex TW11 0LW, UK.; 9 Oncology Safety, Clinical Pharmacology and Safety Sciences, R&D, AstraZeneca, Cambridge CB4 0WG, UK.; 10 Chemistry Research Laboratory, University of Oxford, Mansfield Road, Oxford OX1 3TA, UK.; 11 Institute of Cancer Sciences, University of Glasgow, Glasgow G61 1QH, UK.; 12 Department of Surgery and Cancer, Faculty of Medicine, Imperial College London, Sir Alexander Fleming Building, South Kensington Campus, London SW7 2AZ, UK.; 13 Department of Neurogenetics, Max Planck Institute for Experimental Medicine, Hermann-Rein-Strasse 3, D-37075 Goettingen, Germany.; 14 Natural Products Metabolomics Group, Strathclyde Institute of Pharmacy and Biomedical Sciences, University of Strathclyde, Glasgow G4 0RE, UK.

## Abstract

Alterations to the gut microbiome are associated with various neurological diseases, yet evidence of causality and identity of microbiome-derived compounds that mediate gut-brain axis interaction remain elusive. Here, we identify two previously unknown bacterial metabolites 3-methyl-4-(trimethylammonio)butanoate and 4-(trimethylammonio)pentanoate, structural analogs of carnitine that are present in both gut and brain of specific pathogen–free mice but absent in germ-free mice. We demonstrate that these compounds are produced by anaerobic commensal bacteria from the family Lachnospiraceae (Clostridiales) family, colocalize with carnitine in brain white matter, and inhibit carnitine-mediated fatty acid oxidation in a murine cell culture model of central nervous system white matter. This is the first description of direct molecular inter-kingdom exchange between gut prokaryotes and mammalian brain cells, leading to inhibition of brain cell function.

## Teaser

Discovery of novel bacterial metabolites reveals an unprecedented role for the microbiome in gut-brain axis communication.

## INTRODUCTION

While the microbiome can exert a degree of protection against invading pathogens, substantial and lasting changes in its composition are linked to many diseases that until recently were thought to be independent of microbial influence. Recent work has demonstrated that the microbiome is also fundamentally changed in many neurological conditions including multiple sclerosis, Parkinson’s disease, autism spectrum disorders (ASDs), Alzheimer’s disease, and chronic fatigue syndrome (*1*, *2*). Although little conclusive evidence supports a causative role for the microbiome in neurological disease, bidirectional communication between the gut microbiome and the brain is now recognized as an important mediator of neurological health. Studies in animal models underscore the importance of a stable microbiome, as its disruption, particularly at key developmental stages, results in long-term effects on anxiety, development, and behavior (*3*). In addition, positive effects of gut bacterial supplementation on the brain are reported, while the positive outcomes of therapeutic interventions in some cases can be attributed to the gut microbiota (*4*).

Microbes stimulate host neurotransmitter production in the gut and synthesize their own host-recognized neurotransmitters (*5*, *6*). They can also stimulate the host immune system, leading to inflammation and notable shifts in composition of the gut microbiota with the potential to exert neurological effects (*7*). While microbial-derived molecules such as short-chain fatty acids can cross into the brain to exert their effects, most microbial molecules that influence the microbiome-gut-brain (MGB) axis mediate their effects indirectly through the highly enervated gut and the vagus nerve (*8*, *9*).

## RESULTS

**Fig. 1:**
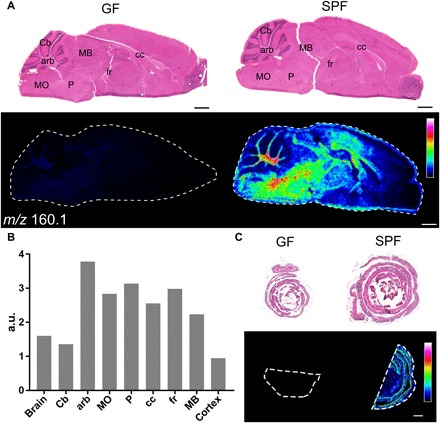
MALDI-MSI on brain and gut sections from C57BL/6 GF and SPF mice. (**A**) Hematoxylin and eosin-stained sections (top) and MALDI-MSI images (bottom) of brain tissue. MALDI-MSI identified a peak at *m/z* 160.133 that was absent in GF mice but present in discrete locations in the brains of SPF mice. (**B**) Bar plot of relative abundance of *m/z* 160.1 in different regions of the SPF brain and the average across the whole brain. (**C**) This metabolite was also present in the SPF colon but absent in the GF colon. a.u., arbitrary units. Annotated brain regions: Cb, cerebellum; arb, arbor vitae; MO, medulla oblongata; P, pons; MB, midbrain; cc, corpus callosum; fr, fasciculus retroflexus. The heatmap intensity bar shows the color scale from low levels of the molecule (black/dark blue) to high levels of the molecule (pink/white). Scale bars, 1 mm.

To identify unknown microbial metabolites that mediate MGB axis communication, we used mass spectrometry imaging (MSI) to identify molecules that are present in both the gut and brain of specific pathogen–free (SPF) C57BL/6 mice but are absent from germ-free (GF) C57BL/6 mice. This approach enables the identification of both known and novel molecules, with high spatial resolution. We detected a molecule at a mass-to-charge ratio (*m/z*) of 160.133 by matrix-assisted laser desorption/ionization (MALDI)–MSI and desorption electrospray ionization (DESI)–MSI in the gut and brain of SPF mice at levels greater than 20 times higher than in corresponding tissue sections from GF mice (Fig. 1 and fig. S1). MALDI-MSI was used to gain a high lateral resolution image for brain region analysis, and DESI-MSI was used for further high spectral resolution analysis. The detection of the molecule at *m/z* 160.133 in GF mice was comparable to a baseline off tissue negative control (fig. S1). This molecule, provisionally termed Met1, was particularly abundant in white matter areas of the brain (medulla, corpus callosum, and the arbor vitae) (Fig. 1B). Met1 was also detected systemically in wild-type C57BL/6 mice, in blood, liver, kidney, lung, spleen, intestine, testes, and heart (fig. S2).

**Fig. 2:**
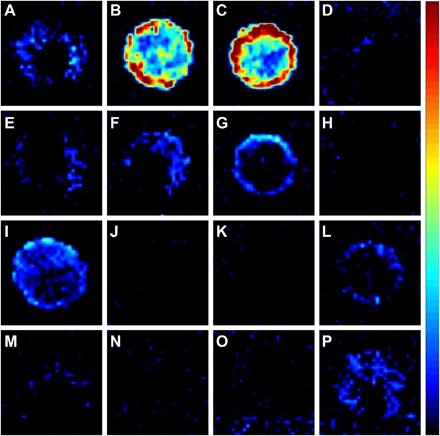
DESI-MSI was used to screen intestinal bacteria for the production of metabolites of interest. Bacterial cultures were grown on solid FAB agar, and colonies were resuspended in PBS to OD_600_ (optical density at 600 nm) of approximately 0.3. Two microliters was allowed to air-dry on a slide before MSI. Bacterial strains tested: (**A**) *C. symbiosum* LM19R, (**B**) *C. symbiosum* LM19B, (**C**) *C. clostridioforme* LM41A, (**D**) *C. symbiosum* LM42D, (**E**) *Bifidobacterium animalis* LM33, (**F**) *Lactobacillus animalis* LM31, (**G**) *Propionibacterium* spp. YM23, (**H**) *Clostridium difficile* LM27, (**I**) *Enterococcus faecalis* YM13, (**J**) *Bacteroides fragilis* NCTC 9343 (type strain), (**K**) *C. clostridioforme* NCTC 11224 (type strain), (**L**) *C. clostridioforme* NCTC 7155 (type strain), (**M**) blank control, (**N**) blank control, (**O**) *C. symbiosum* LM19R, and (**P**) *Escherichia coli* F18.

As detection of Met1 in GF mice was comparable to the negative control, this suggested a microbial origin for the compound in SPF mice. Therefore, we determined whether Met1 in the colon could be reduced by treatment for 7 days with a cocktail of nonabsorbable antibiotics. This treatment was seen to significantly reduce Met1 levels (*P* ≤ 0.05), underscoring the likely microbial origin of Met1 (fig. S3). We next screened intestinal bacterial strains from the gut microbiota of C57BL/6 mice for the production of Met1. Bacterial strains isolated from murine feces were grown on fastidious anaerobe broth (FAB) agar plates before colonies were resuspended in phosphate-buffered saline (PBS) and spotted onto glass slides for screening for Met1 by MSI. A peak at *m/z* 160.133 was detected in two closely related bacterial strains from the *Clostridium* XIVa cluster of the Lachnospiraceae family, determined by 16*S* ribosomal DNA (rDNA) sequencing to be *Clostridium clostridioforme* and *Clostridium symbiosum* (Fig. 2). Both strains are enteric spore-forming human gut commensals that are obligately anaerobic, while *C. clostridioforme* is also an opportunistic pathogen (*10*).

**Fig. 3:**
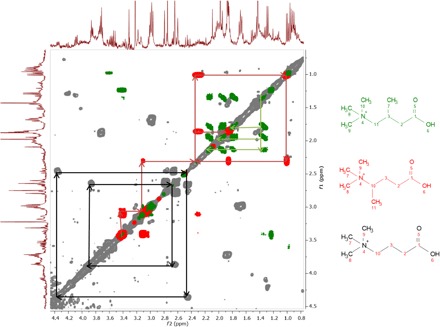
^1^H-^1^H COSY of crude extracts showing the occurrence of three related trimethylammonium derivatives. Cross peaks have been colored-coded accordingly, while lines shown on the spectrum represent the correlation between neighboring coupling protons in the respective derivative: 3M-4-TMAB (green) and 4-TMAP (red) as well as the demethylated congener, GBB (black).

Other clostridial strains tested here did not produce this molecule, and its production was not detected in all *C. clostridioforme* strains tested, including the *C. clostridioforme* type strain NCTC11224 (Fig. 2). To ensure that the bacterial metabolite was Met1, rather than a structural isomer, we carried out tandem mass spectrometry (MS/MS) analysis of the bacterial metabolite at *m/z* 160.133 from *C. clostridioforme* and Met1 from the murine brain. The Met1 product ion spectra from the different sources matched, confirming that the molecules from the murine brain and *C. clostridioforme* were structurally identical (fig. S4). On the basis of the acquired MS/MS spectra, we concluded that Met1 is similar in structure to carnitine, as two indicative mass fragments for a trimethylamine group were observed (*m/z* 58.066 and *m/z* 60.081) along with the mass difference of a carboxylic acid group (the difference between product ions at *m/z* 101.060 and *m/z* 55.055). Furthermore, the MS/MS spectra generated appeared highly similar to that previously reported for 5-aminovaleric acid betaine (5-AVAB; also known as *N*-trimethyl-5-aminovalerate), which has an identical *m/z* 160.133 (*11*). However, during MS/MS fragmentation, we noted distinct differences in the intensity of the peaks at *m/z* 60.081 and *m/z* 55.055 at higher collision energies between Met1 and a synthesized 5-AVAB standard, leading us to doubt that Met1 was 5-AVAB (fig. S5). To definitively elucidate the structure, we subjected the bacterial fraction containing Met1 to nuclear magnetic resonance (NMR) correlation spectroscopy (^1^H-^1^H COSY). NMR determined that Met1 was a mixture of two structural isomers differing only in the location of a methyl side chain, located on either C3 [3-methyl-4-(trimethylammonio)butanoate (3M-4-TMAB)] or C4 [4-(trimethylammonio)pentanoate (4-TMAP)] of the structure (Fig. 3). NMR analysis also showed that the metabolites were produced in an equimolar ratio by the bacteria alongside the congener molecule γ-butyrobetaine (GBB). Detection of GBB alongside 3M-4-TMAB and 4-TMAP in *C. clostridioforme* suggests that it may be the precursor of these molecules, as well as being the direct precursor of carnitine. However, unlike 3M-4-TMAB and 4-TMAP, GBB is synthesized by a range of intestinal bacteria as well as mammalian cells, meaning that tracking GBB produced specifically by the bacterial producers of 3M-4-TMAB and 4-TMAP was not possible.

**Fig. 4:**
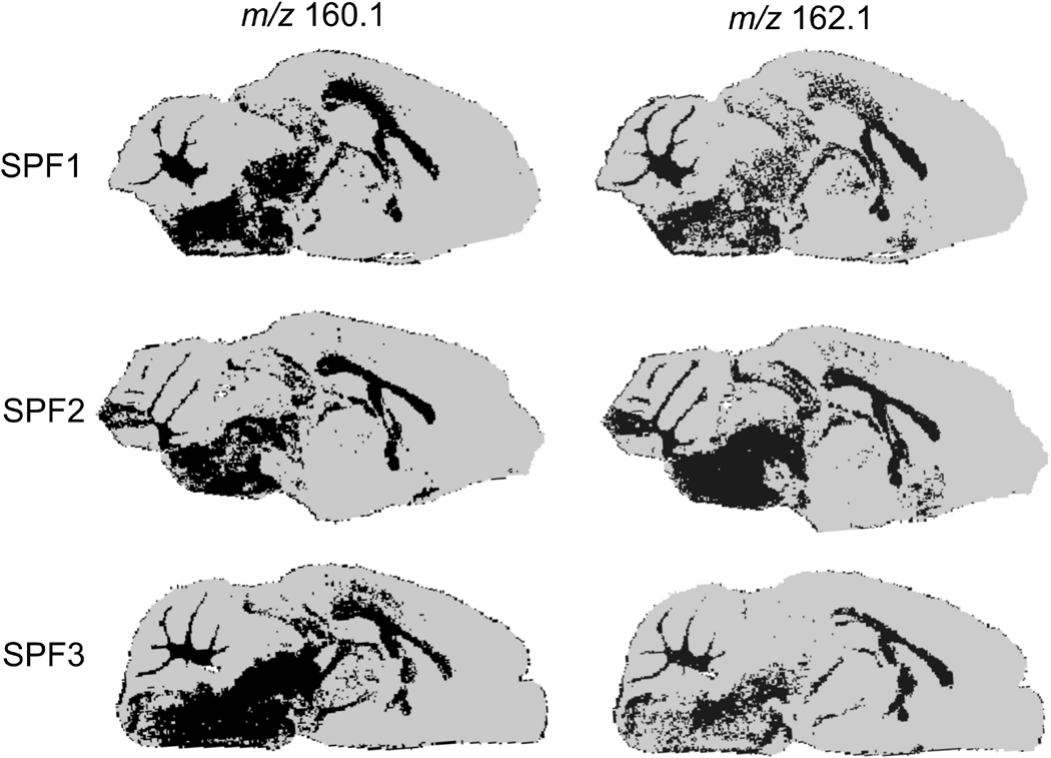
Conversion of MALDI-MSI into binary images showing the high-intensity (black) and low-intensity (gray) regions of the ion images of *m/z* 160.1 (3M-4-TMAB/4-TMAP) and *m/z* 162.1 (carnitine). The overlap of these regions was then calculated as the percentage of pixels in the high-intensity region of the *m/z* 160.1 image that were also high intensity in the *m/z* 162.1 image. In addition, Pearson’s correlation coefficient between the two images was calculated for each tissue across three biological replicates (SPF1, SPF2, and SPF3), with a coefficient of 1 indicating perfect colocalization and −1 indicating no colocalization whatsoever (SPF1 overlap 92.85714, Pearson’s correlation coefficient 0.921547; SPF2 93.20652, 0.906855; SPF3 63.20542, 0.609946).

Because 3M-4-TMAB is highly similar in structure to carnitine and 4-TMAP is similar to GBB, the direct precursor of carnitine, we used MSI to examine colocalization of 3M-4-TMAB and 4-TMAP with carnitine in SPF mouse brain slices. Carnitine is a crucial mediator of fatty acid oxidation (FAO), shuttling long-chain fatty acids in the form of acylcarnitines across the membrane into mitochondria where they are broken down. Carnitine visualization was performed using MALDI-MSI, and DESI-MSI was used to gain an accurate *m/z* (162.112) for subsequent identification (Fig. 4). Mass spectra from MALDI-MSI and DESI-MSI of *m/z* 160.1 and *m/z* 162.1 are shown in fig. S6. Processing of MSI images of 3M-4-TMAB/4-TMAP and carnitine was performed using SpectralAnalysis (*12*). The overlap of high-intensity regions of *m/z* 160.1 (3M-4-TMAB and 4-TMAP) and *m/z* 162.1 (carnitine) was calculated, and the Pearson’s correlation coefficient was determined for these molecules across three sections from each of three SPF mice. The results indicate significant overlap between the signals and spatial colocalization between 3M-4-TMAB and 4-TMAP and carnitine in the brain (Pearson’s correlation: SPF1, 0.921547; SPF2, 0.906855; SPF3, 0.609946), alongside a number of other molecules that had a Pearson’s correlation coefficient over 0.8 (Fig. 4). The Pearson’s correlation coefficient was also determined for *m/z* 160.1 and *m/z* 162.1 in GF mouse brains, and the presence of these was found not to correlate (Pearson’s correlation: GF1, −0.12976; GF2, −0.00027; GF3, 0.177441; fig. S6). This further suggests that the signal at *m/z* 160.1 detected in the GF mouse brain could result from molecules of similar mass (as described in fig. S1B) that could not be resolved in the present study. To ensure that molecular colocalization in SPF mice was underlying the correlation, as opposed to tissue-based changes in ion suppression, total ion count normalization was also applied before analysis. This indicated that carnitine at *m/z* 162 is in the top 3 highly correlated molecules with 3M-4-TMAB/4-TMAP (table S1). Therefore, as well as being structurally similar to carnitine and its precursor, 3M-4-TMAB and 4-TMAP were also found in the same locations within the brain.

**Fig. 5:**
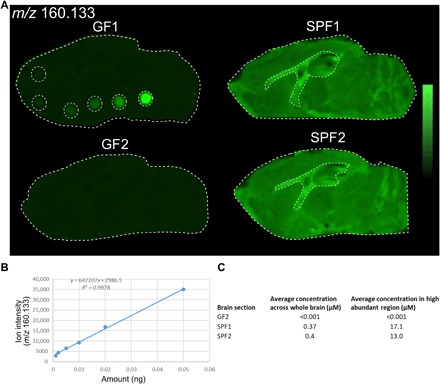
Quantitation of 3M-4-TMAB and 4-TMAP in the mouse brain was performed using DESI-MSI of *m/z* 160.133. (**A**) An equimolar mix of 3M-4-TMAB and 4-TMAP standards was prepared and spotted on a GF brain section at various concentrations. (**B**) The *m/z* 160.133 ion intensity of each spot from the MSI results was used to generate a concentration curve against the amount of standard in each spot. (**C**) The concentration curve was used to calculate the average endogenous concentration of 3M-4-TMAB and 4-TMAP across the whole brain and across the high abundance area, the corpus callosum, and the hippocampus region. The high abundant region is outlined in (A). The results show two technical replicates for GF and SPF brain sections.

Following the structural characterization of the 3M-4-TMAB and 4-TMAP metabolites, standards of each were synthesized and used to quantify the endogenous concentration in the SPF brain through MSI. 3M-4-TMAB and 4-TMAP were determined to be present at equimolar levels in bacterial samples by NMR (Fig. 3); therefore, standards of 3M-4-TMAB and 4-TMAP were equally mixed, and a concentration curve was generated by spotting defined concentrations of this standard on GF brain before MSI analysis. This was used to determine the concentration of the endogenous metabolites in the SPF brain. The average concentration across the whole brain of the metabolites was determined to be 0.37 to 0.4 μM. The average concentration across the corpus callosum and hippocampus was higher, around 13 to 17.1 μM, indicating a notable accumulation of 3M-4-TMAB/4-TMAP in these areas (Fig. 5 and fig. S7).

**Fig. 6:**
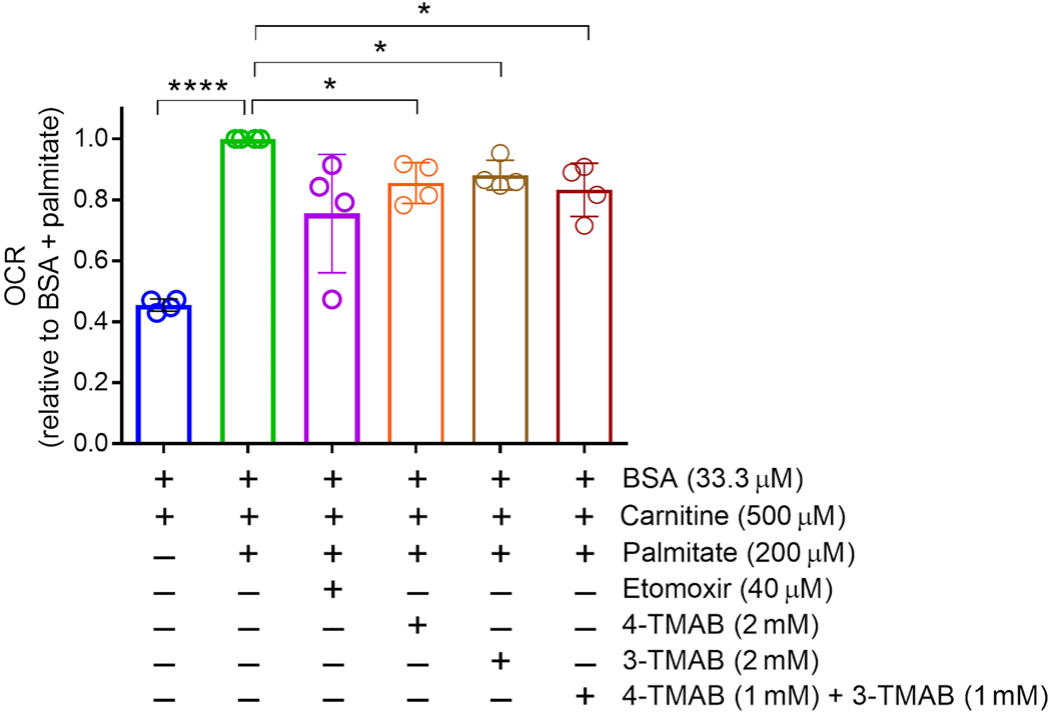
OCR was used as an indicator of FAO in the presence of 3M-4-TMAB and 4-TMAP. Oxidation of palmitate is significantly decreased in primary murine myelinating CNS white matter cultures in the presence of carnitine and either 3M-4-TMAB, 4-TMAP, or a combination of 3M-4-TMAB and 4-TMAP at the indicated concentrations. Etomoxir, an irreversible inhibitor of FAO, was used as a control. The OCR values shown are relative to the BSA and palmitate condition. Each symbol represents the relative mean value for one experiment (*n* = 4 independent experiments). The neural precursors used to generate the model of CNS white matter were obtained by pooling dissociated spinal cord cells from all embryos from a single pregnant female mouse. A one-sample *t* test was used to test the statistical significance against the relative value 1. *P* values ≤0.05 were considered significant. *P < 0.05 and ****P < 0.0001. Bars represent mean ± SD.

Considering the level of structural similarity and spatial colocalization that was observed between bacterial 3M-4-TMAB and 4-TMAP and host carnitine, we hypothesized that these bacterial molecules may inhibit carnitine function in host cells. Using synthetic 3M-4-TMAB and 4-TMAP, we tested their ability to inhibit mitochondrial function in the presence of carnitine in a primary murine cell culture model of central nervous system (CNS) white matter (*13*) using the XF palmitate–bovine serum albumin (BSA) FAO substrate on a Seahorse analyzer (Seahorse Bioscience). This assay measures FAO using the oxygen consumption rate (OCR) as a readout, therefore allowing us to evaluate whether a molecule is capable of inhibiting FAO. Murine spinal cord–derived cells were grown for 8 days in vitro before being assayed in the presence of 0.5 mM carnitine and 3M-4-TMAB and 4-TMAP, either individually or in combination, at 2 mM for 24 hours. FAO was significantly inhibited by 3M-4-TMAB alone (reduced by 12% across four biological replicates), by 4-TMAP (14% reduction), or by an equimolar mixture of 3M-4-TMAB/4-TMAP (17% reduction) (Fig. 6). The inhibition seen is comparable to that for etomoxir (25% reduction), an irreversible inhibitor of FAO. In addition, testing 3M-4-TMAB and 4-TMAP in combination at 20 μM, in the absence of carnitine supplementation into culture and assay media, indicated that 3M-4-TMAB and 4-TMAP, at concentrations similar to those found in the murine brain, could significantly reduce FAO in white matter cells (fig. S8).

The application of a multidisciplinary approach and novel imaging methodologies here has enabled the elucidation of molecular mimicry as another means of communication across the MGB axis. Microbe-mitochondria cross-talk may be an important means of gut microbiome–host communication, and its biological importance in diseases where mitochondrial dysfunction and the gut microbiome are significant factors is likely underappreciated.

## DISCUSSION

Alterations in the gut microbiome have been linked to a number of neurological conditions, meaning that understanding the means of microbial communication across the MGB axis is of increasing importance. This study identifies two novel gut microbiome–derived carnitine mimics that significantly colocalize with carnitine in the white matter of the murine brain while also inhibiting carnitine function in in vitro models of murine CNS white matter. FAO supplies up to 20% of brain oxidative energy needs and is critical for many neurological functions, with neural stem cells reliant in vitro and in vivo on FAO for their survival and proliferation (*14*, *15*). Given recent findings underlining the fundamental importance of FAO to brain health, the identification of gut microbiome–derived compounds in the brain that mediate inhibition of brain cell function is of immense importance (*15*, *16*).

Inhibition of mitochondrial function by antimicrobials has been reported previously, and there is a growing body of evidence to support the existence of microbiome-mitochondria communication within the host (*17*). While carnitine is not needed for fatty acid trafficking in microbes, it acts as both a nutrient source and a protectant against environmental stress (*18*). Carnitine metabolism by enteric microbes generates trimethylamine *N*-oxide (TMAO), high serum levels of which are indicative of cardiovascular disease risk (*19*). Intestinal bacteria can thrive in the presence of carnitine, but *Lachnospiraceae*, of which the 3M-4-TMAB– and 4-TMAP–producing *Clostridia* XIV are members, decrease in its presence likely as a result of being outcompeted by carnitine-metabolizing bacteria (*19*). Under such competitive conditions, we speculate that carnitine mimics such as 3M-4-TMAB and 4-TMAP may be produced by these *Lachnospiraceae* in an attempt to inhibit carnitine function or metabolism in competing bacteria.

*C. clostridioforme* and *C. symbiosum*, the identified producers of 3M-4-TMAB and 4-TMAP, remain poorly understood. *C. clostridioforme* is now recognized as distinct from *C. bolteae* and *C. hathewayi*, two species that were previously classified as *C. clostridioforme* (*10*). *C. bolteae* rapidly proliferates after disruption of the human gut microbiota, likely because of its ability to form endospores and resistance to multiple antibiotics, traits also found in *C. clostridioforme* and *C. symbiosum* (*20*, *21*). *C. bolteae* exhibits a marked ability to proliferate and persist in the human intestine post-antibiotic use, maintaining its presence for 180 days after treatment (*20*). The presence of *C. symbiosum* and *C. clostridioforme* in the gut microbiome is associated with low microbial diversity, while *C. symbiosum* presence could further distinguish obese from lean participants (*22*). The link between *C. symbiosum* and metabolism is further underlined by its ability to promote robust weight gain in growth-stunted mice, one of only two bacteria that exhibited the capability (*23*). Whether 3M-4-TMAB and 4-TMAP could play any role in these metabolic effects warrants further investigation, but we hypothesize that under conditions that are favorable to increased colonization by these microbes, the production of 3M-4-TMAB and 4-TMAP in the intestine is likely to increase significantly with potential systemic effects.

While 3M-4-TMAB is a molecule not previously described in the literature, 4-TMAP has been chemically synthesized as an analog of mildronate, a potent inhibitor of both human GBB hydroxylase (BBOX1) and carnitine acetyltransferase (CrAT) enzymes (*24*). These enzymes are involved in carnitine synthesis and the transport of fatty acids into mitochondria, respectively. 4-TMAP is bound in the human BBOX1 active site in a near-identical conformation to mildronate and has a lower dissociation constant (4.1 μM) but was found to have an inhibitory concentration (IC_50_) nearly three orders of magnitude higher than mildronate (*24*–*26*). This means that the inhibitory effects seen here are likely mediated by targeting of carnitine-related enzymes other than BBOX1 and CrAT. 3M-4-TMAB bears marked structural similarity to a number of known inhibitors of carnitine function, including aminocarnitine derived from the fungal compound emericidin (*27*). Aminocarnitine inhibits carnitine palmitoyltransferase 1 (CPT1) and CPT2, and CrAT as the hydroxyl group essential to carnitine metabolism has been substituted with an alternate nonfunctional side chain. Removal of this hydroxyl group, which is also absent in 3M-4-TMAB and replaced with a methyl group, prevents the esterification reaction needed to link a long-chain fatty acid to carnitine, thereby inhibiting fatty acid shuttling into the mitochondria for oxidation. Future research focusing on a role for metabolites from the gut microbiome in the inhibition of mitochondrial function across a wide range of mammalian tissues and cell types may be highly informative. Such work has the potential to yield significant insight into how the gut microbiome influences the host systemically, not just across the MGB axis.

While difficult to determine without further detailed confirmatory tests, the presence of 3M-4-TMAB and 4-TMAP in other diseases is suggested through publicly available data and the literature. A molecule of the exact *m/z* of 3M-4-TMAB and 4-TMAP (160.133) has been described in the literature as a biomarker in type 2 diabetes (T2D), significantly increased in cord plasma during preeclampsia, and predictive of diabetic nephropathy in T1D patients (*28*–*30*). These diseases all have associated mitochondrial dysfunction or incomplete FAO. The likelihood of the biomarker at *m/z* 160.133, which is significantly increased in T2D patients, being 3M-4-TMAB and 4-TMAP, is further supported by separate studies in Europe and China, in which the 3M-4-TMAB/4-TMAP–producing strains were the most significantly enriched species in the T2D gut microbiome (*31*, *32*). *C. symbiosum* has shown promise as a biomarker for early and noninvasive detection of colorectal cancer, while a molecule of *m/z* 160.1 was detected as one of the three most significantly correlated molecules to hypoxic regions in breast cancer tumors (*33*, *34*). The product ion spectra produced from the MS/MS analysis of the metabolite in the breast cancer tumor model match the spectra from 3M-4-TMAB and 4-TMAP, indicating that they are likely the same and that 3M-4-TMAB/4-TMAP can penetrate tumors.

The interplay between the microbiome and host mitochondria is likely far more extensive and complex than presently known, with many diseases of mitochondrial dysfunction correlated to well-described changes in microbial populations in the gut (*17*, *31*, *35*). Mitochondrial dysfunction is also common across a range of neurological conditions, with neuronal cells highly dependent on dynamic mitochondria to meet their high energy requirements (*16*, *36*, *37*). A number of ASDs have been linked to underlying mitochondrial dysfunction, with descriptions in the literature describing increased acylcarnitine levels, decreased FAO-mediated stem cell generation, and carnitine-mediated improvement in symptoms (*16*, *38*). While the significance of alterations in the gut microbiota in ASD remains controversial, both of the 3M-4-TMAB/4-TMAP–producing strains identified here have been described in elevated numbers in the gut while being at low levels or completely absent in controls (*39*). These microbiome changes are mirrored in murine ASD models such as the maternal immune activation (MIA) model, with increases in Lachnospiraceae again noted (*4*). After treatment decreased Lachnospiraceae numbers in the intestine of the MIA model, improvements in communicative, stereotypic, anxiety-like, and sensorimotor behaviors were all noted, along with a correction in defective fatty acid metabolism. These mitochondrial deficiencies, alongside those described in other neurological conditions such as Parkinson’s and Alzheimer’s disease, mean that any potential role for microbiome input into mitochondrial inhibition is worthy of further investigation.

This is the first mechanistic description of a microbial molecule inhibiting the function of the mitochondria in cells of the CNS. The two novel molecules produced by the gut microbiome described here are the first found in the murine brain that localize with and antagonize the function of carnitine. Given their potency at the physiological concentrations found in the murine brain, our findings indicate that neurological conditions, where mitochondrial dysfunction has been described and where disturbances in the gut microbiome are noted, should be looked at with increased emphasis on potential for microbiome input.

## MATERIALS AND METHODS

### Animal work

Seven- to 8-week-old male C57BL/6J GF and SPF mice were sourced from the University of Manchester, Gnotobiotic Facility. Both GF and SPF mice were fed the same pelleted diet, which was sterilized by irradiation with 50 kGy. The Manchester Gnotobiotic Facility was established with the support of the Wellcome Trust (097820/Z/11/B) using founder mice obtained from the Clean Mouse Facility, University of Bern. For determination of bacterial metabolites in organs other than the brain and to determine the effects of antibiotic treatment on the production of 3M-4-TMAB/4-TMAP in the intestine, C57BL/6J mice that were age- and sex-matched to GF mice were sourced from the University of Glasgow. For antibiotic treatment, mice were administered gentamicin (1 mg/ml), neomycin (1 mg/ml), and vancomycin (0.5 mg/ml) in sterile distilled drinking water for 7 days. Approval for these procedures was given before their initiation by internal University of Manchester and University of Glasgow ethics committees and the U.K. Home Office under licenses 70/7815, P64BCA712, and P78DD6240.

### Tissue processing

Mice were culled by cervical dislocation, and brains and colons were immediately dissected. The colons were cut along the length, fecal matter was removed, and the colon was rolled and embedded in 2.5% carboxymethyl cellulose (CMC) (Sigma-Aldrich). Brains were placed unembedded in a mold before freezing using crushed dry ice and ethanol. The testis, heart, lung, liver, kidney, spleen, and mesenteric lymph nodes were collected from one C57BL/6J mouse, embedded in 2.5% CMC and snap-frozen in crushed dry ice and ethanol. Blood was collected by cardiac puncture and spotted onto a glass slide and allowed to air dry. Both brains and colons were cut using a cryostat microtome (Leica) at 10 μm thickness at −18°C, and the sections were thaw-mounted onto indium tin oxide–coated slides for MALDI-MSI and normal glass slides for DESI-MSI. Consecutive brain and gut sections next to those taken for MSI were used for hematoxylin and eosin staining. All slides were stored at −80°C until analysis.

### Matrix application and MALDI-MSI analysis

MALDI was used for the initial analysis to obtain a high lateral resolution image to show an accurate distribution of the metabolite in the brain. Extensive further analysis was performed using high mass resolution DESI analysis for MS/MS experiments, showing that the spectra from the metabolites in the SPF brain and the spectra produced by the bacteria are highly similar. The increase in mass resolution is demonstrated in fig. S9. MSI of brains and guts from GF and SPF mice was carried out by MALDI–time-of-flight (TOF). The results were confirmed by MSI with a DESI-orbitrap to gain a more accurate *m/z* value. Before matrix application, the slide was taken from −80°C and brought to room temperature under a stream of air. α-Cyano-4-hydroxycinnamic acid (CHCA) matrix was applied at a concentration of 5 mg/ml in 50% acetonitrile, 50% water with 0.1% trifluoracetic acid using an automated matrix applicator (HTX Technologies) for eight passes at 75°C, 414 millibar gas pressure, 80 μl/min flow, and 1100 mm/min. The MALDI-MSI experiments were carried out on a rapifleX MALDI-TOF instrument (Bruker Daltonics) with a 10-kHZ smartbeam laser. Imaging was performed at a spatial resolution of 50 μm with 600 laser shots per position. Results were analyzed using flexImaging 5.0 software, and the data were normalized by total ion count.

### DESI-MSI analysis

DESI-MSI was performed on an orbitrap mass spectrometer (Q Exactive, Thermo Fisher Scientific) equipped with an automated Prosolia 2D DESI source (Prosolia OmniSpray 2D). The DESI source was modified with the following parameters. The spray tip was positioned at 1.5 mm above the sample surface and at an angle of 75°. The distance between the sprayer to mass spectrometer inlet was 7 mm with a collection angle of 10°. The spray solvent was methanol/water (95:5, v/v), delivered at 1.5 μl/min using a Dionex Ultimate 3000 pump (Thermo Fisher Scientific) at a spray voltage of 4.5 kV. Nitrogen was used as the nebulization gas at a pressure of 7 bars. The Q Exactive mass spectrometer was operated using an S-Lens setting of 50 V and using *m/z* range of 65 to 400 in positive ionization mode, with a spatial resolution of 100 μm for tissue sections and 150 μm for the blood spot. For acquisition of MS/MS spectra, an injection time of 300 ms, automatic gain control target of 5,000,000, and mass resolution of 70,000 were used. MS/MS analysis was performed using various high collision dissociation settings (shown in Results) and a mass isolation window of ±0.3 Da. Data were converted into imzML format using imzML converter version 1.1.4.5, and data were visualized using MSiReader version 0.09 (*40*).

### MSI analysis of bacterial spots

To isolate producing bacteria from the murine microbiome, feces was isolated from C57BL/6J mice and plated onto FAB agar plates. Single isolated colonies of murine gut microbiome bacteria or type strains were grown overnight in FAB or on FAB agar. Taxonomic assignment for the strains was obtained using the complete 16*S* rDNA sequence from each strain and the “Identify” function in EzBioCloud (*41*). The 3M-4-TMAB/4-TMAP–producing strain, designated LM19, was identified as *C. symbiosum*, being 99.79% similar to the *C. symbiosum* type strain ATCC14940. The second producing strain, designated LM41, was identified as being 99.5% similar to the *C. clostridioforme* type strain ATCC25537. Type strains used for MSI were from the National Collection of Type Cultures (United Kingdom). For MSI analysis, single colonies from FAB agar plates were inoculated into 100 μl of PBS to give an optical density at 600 nm of 0.3. Two microliters of each bacterial culture was spotted onto a glass slide and allowed to dry. Slides were stored in a desiccator until DESI-MSI, which was performed using the same DESI-MSI parameters as above with a spatial resolution of 200 μm.

### Nuclear magnetic resonance

Samples (15 mg) were dissolved in 700 μl of deuterated water (Sigma-Aldrich) and added to a 7-mm NMR tube. Presaturation proton NMR experiments were performed by irradiating the water peak at 4.88 parts per million (ppm), using 128 scans, 15-Hz spin with a relaxation delay of 3 s. NMR was carried out using a 400-MHz JEOL LA400 FT-NMR spectrometer system equipped with a 40TH5AT/FG probe (JEOL, Tokyo, Japan). The acquisition of the one-dimensional proton spectra (^1^H NMR) was performed by the presaturation pulse sequence using 128 scans per analysis. Subsequently, two-dimensional COSY NMR spectra were also acquired with 64 scans. Data points were collected into a plot using spectral widths of 3.93 kHz for F1 and F2. The presaturation method was used to suppress the solvent signal during acquisition.

### Analysis of correlation of *m/z* 160.133 with carnitine in the brain

Data were first converted to imzML using flexImaging (Bruker Daltonics version 4.1), and processing was performed using SpectralAnalysis. Peaks were picked from a mean spectrum generated without any preprocessing using gradient peak detection, and a data cube was generated by integrating the area under each peak. All data were then exported into MATLAB (version 2017a and statistics and image processing toolbox, The MathWorks Inc., Natick, MA) for further analysis.

Tissue data were first segmented from background using *k*-means clustering (*k* = 2, cosine distance). Following this, the images from *m/z* 160.01 and 162.01 (carnitine) were extracted and segmented into binary masks of high and low intensity using a *k*-means clustering (*k* = 2, Euclidean distance). The overlap of these regions was then calculated as the percentage of pixels in the high-intensity region of the *m/z* 160.1 image that were also high intensity in the *m/z* 162.1 image. In addition, Pearson’s correlation coefficient between the two images was calculated for each tissue.

### Synthesis of 3M-4-TMAB and 4-TMAP

All amino acid starting materials were from UkrOrgSyntez Ltd. The general procedure of Lukevics was used to prepare the materials (*42*). The method of Tars was used for purification (*24*).

4-TMAP [*rac*-4-(trimethylammonio)pentanoate] methyl-*N*,*N*′-diisopropylcarbamimidate (0.500 ml, 2.75 mmol) was added dropwise to a stirred solution of 4-(dimethylamino)pentanoic acid hydrochloride (250 mg, 1.38 mmol) in MeOH (4 ml) at 21°C over a period of 1 min under nitrogen. The resulting solution was stirred at 21°C for 40 hours. The reaction mixture was concentrated to dryness under reduced pressure, and the residue was slurried in water (6 ml) for 1 hour. The precipitated urea was removed by filtration, and the mixture was evaporated to dryness to give a gum. This material was dissolved in water (5 ml) and loaded onto a column packed with 5 ml of Amberlite IRN78 hydroxide form resin, and the column was stoppered for 30 min before being eluted with water. Product-containing fractions were combined and evaporated under reduced pressure, and the resulting residue was evaporated from acetonitrile to give *rac*-4-(trimethylammonio)pentanoate (204 mg, 1.281 mmol, 93%) as a white solid. ^1^H NMR (500 MHz, d_6_-DMSO) 1.23 (d, *J* = 6.54 Hz, 3H), 1.29 to 1.39 (m, 1H), 1.76 (ddd, *J* = 6.0, 9.5, 15.5 Hz, 1H), 1.93 (ddd, *J* = 5.5, 6.45, 15.0 Hz, 1H), 2.1 to 2.18 (m, 1H), 2.98 (s, 9H), and 3.43 (dqd, *J* = 2.5, 6.5, 10.5 Hz, 1H); ^13^C NMR (126 MHz, D_2_O, 27°C) 180.9, 71.0, 50.6, 34.2, 26.4, and 12.7; liquid chromatography (LC)–MS: *m/z* 160; assay: 79% (w/w) (quantitative NMR).

3M-4-TMAB [*rac*-3-methyl-4-(trimethylammonio)butanoate] methyl-*N*,*N*′-diisopropylcarbamimidate (0.500 ml, 2.75 mmol) was added dropwise to a stirred solution of 4-(dimethylamino)-3-methylbutanoic acid hydrochloride (250 mg, 1.38 mmol) in MeOH (4 ml) at 21°C over a period of 1 min under nitrogen. The resulting solution was stirred at 21°C for 40 hours. The reaction mixture was concentrated to dryness under reduced pressure, and the residue was slurried in water (6 ml) for 1 hour. The precipitated urea was removed by filtration, and the mixture was evaporated to dryness to give a gum. The material was dissolved into water (5 ml) and loaded onto a column packed with 5 ml of Amberlite IRN78 hydroxide form resin, and the column was stoppered for 30 min before being eluted with water. Product-containing fractions were combined and evaporated under reduced pressure, and the resulting residue was evaporated from acetonitrile to give a gum. The material was sonicated in diethyl ether (ca. 4 ml), and the solvent was removed by pipette to leave a waxy solid that was dried under reduced pressure to give *rac*-3-methyl-4-(trimethylammonio)butanoate (88 mg, 0.553 mmol, 40%) as a waxy gum. ^1^H NMR (500 MHz, D_2_O): 1.18 (d, *J* = 6.5 Hz, 3H), 2.23 (dd, *J* = 7.8, 14.5 Hz, 1H), 2.34 (dd, *J* = 6.5, 14.5 Hz, 1H), 2.41 to 2.51 (m, 1H), 3.18 (s, 9H), 3.29 (dd, *J* = 6.0, 13.5 Hz, 1H), and 3.37 (dd, *J* = 3.54, 13.5 Hz, 1H); ^13^C NMR (126 MHz, D_2_O, 27°C): 180.17, 72.37, 53.38, 44.39, 26.62, and 20.13; LC-MS: *m/z* 160; assay: 74% (w/w) (quantitative NMR).

### 3M-4-TMAB and 4-TMAP quantitation

A stock solution of 3M-4-TMAB and 4-TMAP (AstraZeneca compound management) was prepared daily as needed at a concentration of 1 mg/ml in 50:50 (v/v) methanol/water. The stock solutions (1 mg/ml) were subsequently mixed to form a working solution (0.1 mg/ml), which was then serially diluted to 11 further working solutions with concentrations in the range of 0.00002 to 0.04 mg/ml. Working solutions at 0.00002, 0.00004, 0.0001, 0.0002, 0.0004, and 0.001 mg/ml were spotted (50 nl using a Biospotter, Biofluidix, Freiburg, Germany) onto GF mouse brain tissue and allowed to evaporate. This deposition of standard translated to 0.001, 0.002, 0.005, 0.01, 0.02, and 0.05 ng of 3M-4-TMAB/4-TMAP on tissue. DESI-MSI analysis was performed using the same parameters as above. Data visualization and region of interest extraction were performed using SCiLS Lab MVS 2018b (Bruker Daltonics, Bremen, Germany) software typically using mass selection window of ±0.05 Da. 3M-4-TMAB/4-TMAP was detected as the protonated molecular ion (M^+^H^+^) at *m/z* 160.1. Extracted mean relative abundance was used from each area of interest to construct calibration curves in Microsoft Excel using a linear fit with unknown endogenous concentrations calculated using the equation of a straight line.

### Cell culture

CNS myelinating cultures were established as described (*13*) with minor modifications. Briefly, embryonic day 13 (E13; day of plug E0) mouse spinal cords were isolated and stripped of their meninges and then dissociated into a single-cell suspension using trypsin and trituration. Cells were plated at 60,000 to 75,000 cells per well of a Seahorse XF96 cell culture plate (35-μl volume) pretreated with poly-l-lysine [in boric acid buffer (0.1 mg/ml; pH 8.4)]. Cells were plated initially in 12.5% horse serum, which was gradually withdrawn through feeding every second or third day with serum-free differentiation medium [Dulbecco’s modified Eagle’s medium (DMEM) (glucose, 4.5 mg/ml), penicillin (100 U/ml), streptomycin (100 μg/ml), biotin (10 ng/ml), 1% N1, 50 nM hydrocortisone, and insulin (10 μg/ml)]. Cells were maintained in 5% CO_2_ at 37°C.

### OCR measurements

On day in vitro (DIV) 8 for CNS myelinating cultures, OCR was measured using the XF Cell Mito Stress Test and XF Palmitate-BSA FAO substrate on a Seahorse XF96 analyzer (Seahorse Bioscience, Billerica, MD). Twenty-four hours before the assay, cultures were placed in Substrate-Limited Medium [DMEM supplemented with 0.5 mM glucose, 1 mM glutamine, 0.5 mM carnitine, and 1% fetal bovine serum (FBS)]. Just before the assay, media were changed to FAO Assay Medium consisting of KHB buffer (111 mM NaCl, 4.7 mM KCl, 1.25 mM CaCl_2_, 2 mM MgSO_4_, and 1.2 mM NaH_2_PO_4_) supplemented with 2.5 mM glucose, 0.5 mM carnitine, and 5 mM Hepes, adjusted to pH 7.4. To stimulate FAO, palmitate-BSA substrate (200 μM) was added to all wells except the BSA control. 4-TMAP and 3M-4-TMAB effects on FAO were measured by supplementation into wells of 2 mM 4-TMAP or 3M-4-TMAB or 1 mM 4-TMAP and 1 mM 3M-4-TMAB in combination for 24 hours before the assay and for the duration of the assay. For examination of the effect of the concentrations of 3M-4-TMAB and 4-TMAP found in the brain on FAO, the experiment was repeated as before but in the absence of carnitine (0.5 mM) supplementation into both Substrate-Limited Medium and FAO Assay Medium, meaning that FBS was the only exogenous source of carnitine during the assay. 4-TMAP or 3M-4-TMAB (20 μM), or a combination of 10 μM 4-TMAP and 10 μM 3M-4-TMAB, was supplemented into media for 24 hours before assay and during the assay. OCR was measured over time in the presence of either high or low concentrations of 3M-4-TMAB and 4-TMAP, and at least three repeated measures per well were recorded. For each well, the measurements were normalized on the total micrograms of proteins determined at the end of the assay with a Pierce BCA Protein Assay kit (Thermo Fisher Scientific). The number of replicate wells varied between 5 and 20 per condition. The values of the repeated measurements were averaged, and a mean value was obtained for each individual well. The OCR values per well were further averaged, and a mean value for each condition was obtained. Last, the mean value for each of the conditions was divided by the value obtained for the condition with palmitate and BSA supplementation. These calculations were applied to each of the independent experiments performed, for which the relative OCR values are reported in the figure.

### Microbiome analysis of antibiotic-treated mice

For preparation of DNA samples for microbiome sequencing, fecal samples were defrosted to room temperature and the FastDNA Spin Kit for Soil DNA Extraction (MP Biomedicals) was used for DNA extraction; 16*S* ribosomal RNA (rRNA) gene sequencing was undertaken at Glasgow Polyomics for analysis of the microbiome. In brief, primers that were specific to the V3 and V4 regions were used to generate 16*S* amplicons from 12.5 ng of extracted DNA using polymerase chain reaction. The resulting amplicons had dual barcodes and adapters added using the Nextera XT v2 adapter sets (Illumina). The libraries were combined in equimolar ratios and sequenced on a MiSeq (Illumina) instrument using a paired end, 2 × 300–base pair (bp) sequencing run. Samples were sequenced with an average of 50,000 reads.

For data analysis, FastQ files were generated from the sequencing data and quality-filtered using cutadapt (*43*). Cutadapt removes adapter sequences from high-throughput sequencing reads with a minimum quality of 25 and a minimum length of 250 bp. Paired-end reads were combined using PANDAseq and collated into a single FASTA file using the Qiime package (*44*). Further processing and analysis were completed using the Qiime wrapper and software.

### Statistical analysis

For analysis of FAO, at least three independent experiments were undertaken, with each biological replicate shown. For each of the conditions tested, the value of OCR was expressed as relative to the BSA and palmitate condition, and the significance of the differences between conditions was assessed with a one-sample *t* test (relative value = 1, GraphPad Prism 8.1.2). *P* values ≤0.05 were considered significant for all statistical tests.

## Supplementary Material

aax6328_SM.pdf

## SUPPLEMENTARY MATERIALS

Supplementary material for this article is available at http://advances.sciencemag.org/cgi/content/full/6/11/eaax6328/DC1 Fig. S1. Mass spectra from MALDI-MSI and DESI-MSI experiments with the average spectra from GF and SPF mouse brains overlaid. Fig. S2. MALDI-MSI of organs and blood recovered from C57BL/6 wild-type mice indicated that *m/z* 160.1 was located systemically in all organs tested as well as present in whole blood. Fig. S3. MALDI-MSI of colon sections from mice treated with antibiotics for 7 days demonstrates that the metabolite at *m/z* 160.1 is decreased in the colon of antibiotic-treated mice compared to controls. Fig. S4. MS/MS analysis of the metabolite found in the brain and the metabolite of the same *m/z* (160.133) produced by *C. clostridioforme* was undertaken. Fig. S5. Mass spectra from the MS/MS analysis of the synthesized standard 5-AVAB compared to the endogenous metabolite in the brain at *m/z* 160.133. Fig. S6. Overlap of the ion images of *m/z* 160.1 and *m/z* 162.1 (carnitine) from SPF and GF mouse brains and abundance of *m/z* 160.1 from SPF and GF brains and off-tissue negative control area. Fig. S7. Additional information to fig. S5. Fig. S8. OCR was used as an indicator of FAO in the presence of 3M-4-TMAB and 4-TMAP at the indicated concentrations and in the absence of carnitine supplementation. Fig. S9. Average mass spectra from MALDI-MSI and DESI-MSI results showing peaks at *m/z* 160.133 (3M-4-TMAB/4-TMAP) and *m/z* 162.112 (carnitine) from GF and SPF mouse brains. Table S1. Summary of the top correlations with Pearson’s coefficient > 0.5 for *m/z* 160.1 (3M-4-TMAB/4-TMAP) for the SPF brain tissue sections shown in Fig. 4.

## Appendix

View/request a protocol for this paper from *Bio-protocol*.
