# Supplementary material for: Microbiome-derived carnitine mimics as previously unknown mediators of gut-brain axis communication
Source: Sci Adv. 2020 Mar 11;6(11):eaax6328. doi: 10.1126/sciadv.aax6328 (PMC7065903; doi:10.1126/sciadv.aax6328)
Supplement: aax6328_SM.pdf [file aax6328_SM.pdf]

Science Advances
AAAS

advances.sciencemag.org/cgi/content/full/6/11/eaax6328/DC1

Supplementary Materials for

Microbiome-derived carnitine mimics as novel mediators of gut-brain axis communication

Heather Hulme, Lynsey M. Meikle, Nicole Strittmatter, Justin J. J. van der Hooft, John Swales, Ryan A. Bragg, Victor H. Villar, Michael J. Ormsby, Stephanie Barnes, Sheila L. Brown, Alex Dexter, Maya T. Kamat, Jasper C. Komen, Daniel Walker, Simon Milling, Emily K. Osterweil, Andrew S. MacDonald, Chris J. Schofield, Saverio Tardito, Josephine Bunch, Gillian Douce, Julia M. Edgar, RuAngelie Edrada-Ebel, Richard J. A. Goodwin, Richard Burchmore, Daniel M. Wall*

*Corresponding author. Email: donal.wall@glasgow.ac.uk

Published 11 March 2020, *Sci. Adv.* **6**, eaax6328 (2020)
DOI: 10.1126/sciadv.aax6328

This PDF file includes:

Fig. S1. Mass spectra from MALDI-MSI and DESI-MSI experiments with the average spectra from GF and SPF mouse brains overlaid.
Fig. S2. MALDI-MSI of organs and blood recovered from C57BL/6 wild-type mice indicated that *m/z* 160.1 was located systemically in all organs tested as well as present in whole blood.
Fig. S3. MALDI-MSI of colon sections from mice treated with antibiotics for 7 days demonstrates that the metabolite at *m/z* 160.1 is decreased in the colon of antibiotic-treated mice compared to controls.
Fig. S4. MS/MS analysis of the metabolite found in the brain and the metabolite of the same *m/z* (160.133) produced by *C. clostridioforme* was undertaken.
Fig. S5. Mass spectra from the MS/MS analysis of the synthesized standard 5-AVAB compared to the endogenous metabolite in the brain at *m/z* 160.133.
Fig. S6. Overlap of the ion images of *m/z* 160.1 and *m/z* 162.1 (carnitine) from SPF and GF mouse brains and abundance of *m/z* 160.1 from SPF and GF brains and off-tissue negative control area.
Fig. S7. Additional information to fig. S5.
Fig. S8. OCR was used as an indicator of FAO in the presence of 3M-4-TMAB and 4-TMAP at the indicated concentrations and in the absence of carnitine supplementation.
Fig. S9. Average mass spectra from MALDI-MSI and DESI-MSI results showing peaks at *m/z* 160.133 (3M-4-TMAB/4-TMAP) and *m/z* 162.112 (carnitine) from GF and SPF mouse brains.
Table S1. Summary of the top correlations with Pearson's coefficient > 0.5 for *m/z* 160.1 (3M-4-TMAB/4-TMAP) for the SPF brain tissue sections shown in Fig. 4.

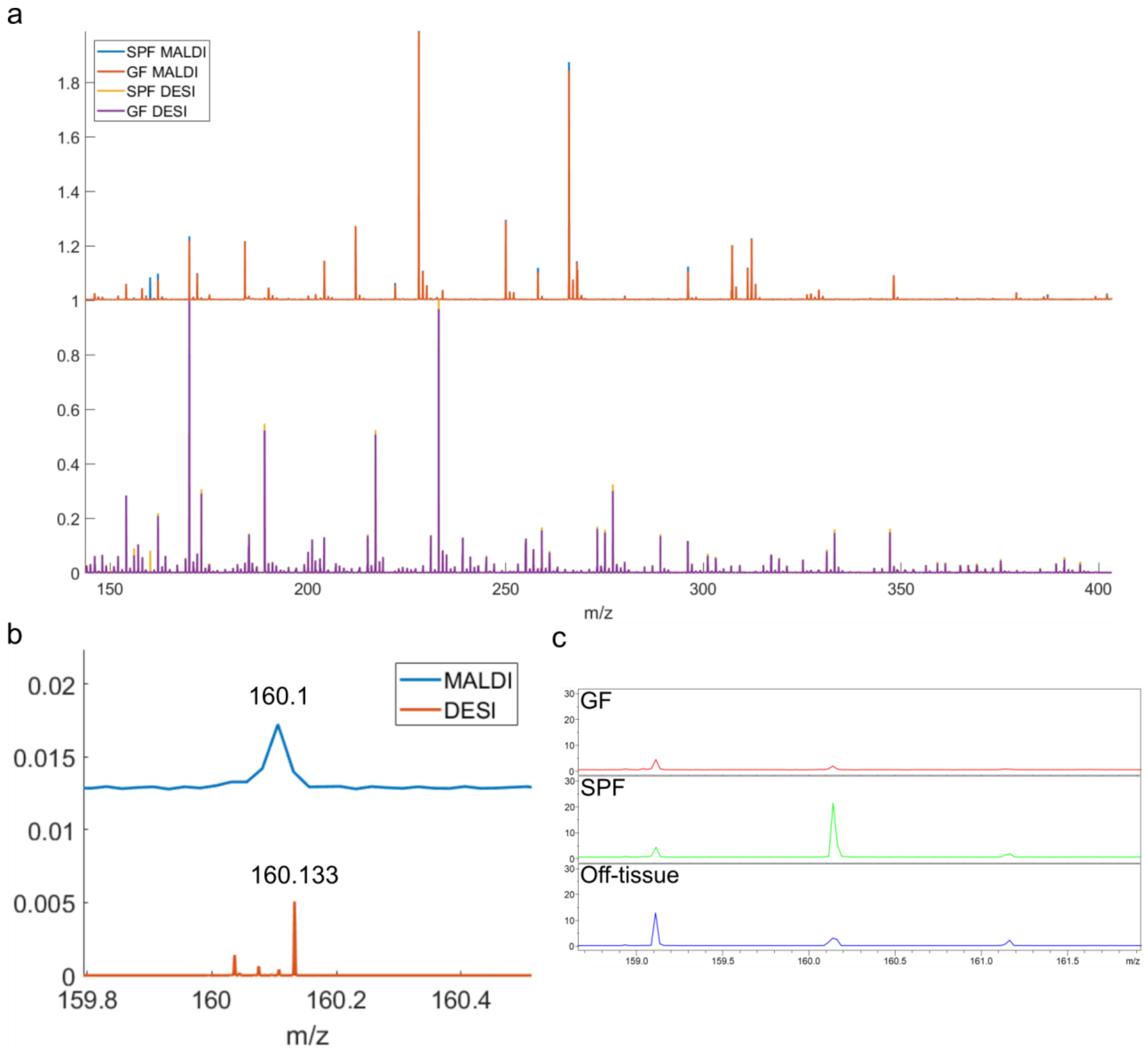

Fig. S1. Mass spectra from MALDI-MSI (top panel, data shown in Figure 1) and DESI-MSI experiments (bottom panel, data shown in Figure 5) with the average spectra from GF and SPF mice brains overlaid (a). The spectra show similar peaks are detected in the GF and SPF mice, with the peak at *m/z* 160.133 not visible in the GF spectra. A zoomed average spectrum from MALDI-MSI and DESI-MSI experiments show the peak at *m/z* 160.133 (b). There are several peaks present in the high-resolution DESI-MSI spectrum, which could not be resolved by MALDI-MSI. These unresolved peaks would contribute to apparent detection for *m/z* 160.1 in

the GF samples. A comparison of MALDI-MSI average spectra from GF brains, SPF brains and a region off-tissue, demonstrate that the *m/z* 160.1 peak from the GF brain is comparable to a spectra acquired from a region off-tissue (c).

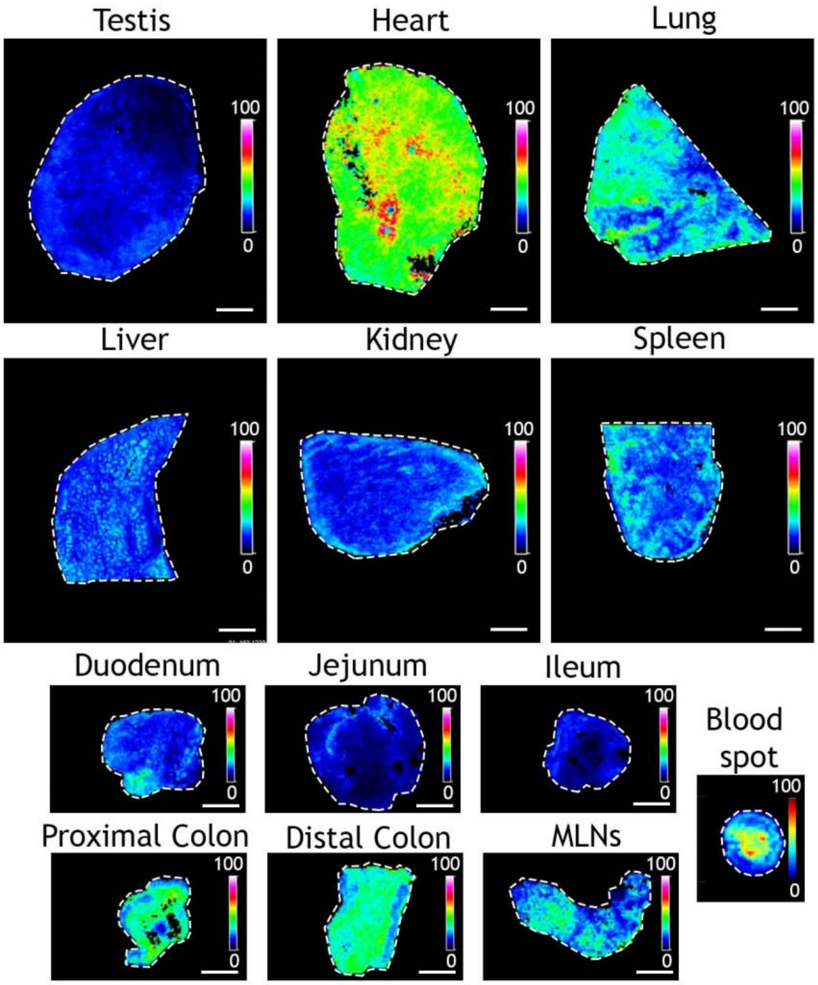

Fig. S2. MALDI-MSI of organs and blood recovered from C57BL/6 wild-type mice indicated that *m/z* 160.1 was located systemically in all organs tested as well as present in whole blood. Levels were lowest in the testis and in the ileum, while highest levels were present in the colon and in the heart tissue.

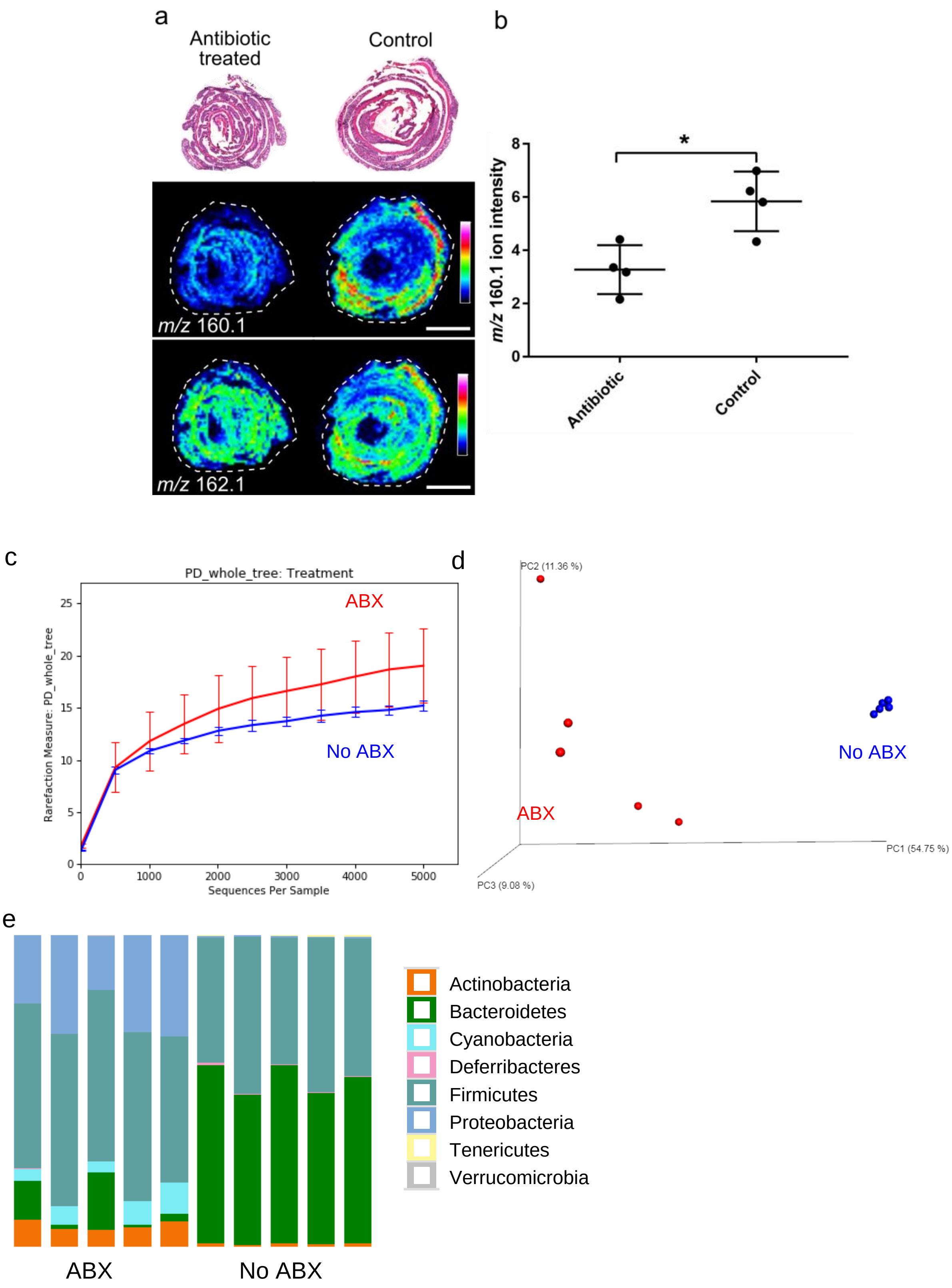

a
Antibiotic treated
Control
m/z 160.1
m/z 162.1
b
*
m/z 160.1 ion intensity
Antibiotic
Control
c
PD_whole_tree: Treatment
ABX
No ABX
Rarefaction Measure: PD_whole_tree
Sequences Per Sample
d
PC2 (11.36 %)
PC1 (54.75 %)
PC3 (9.08 %)
ABX
No ABX
e
Actinobacteria
Bacteroidetes
Cyanobacteria
Deferribacteres
Firmicutes
Proteobacteria
Tenericutes
Verrucomicrobia
ABX
No ABX

Fig. S3. MALDI-MSI of colon sections from mice treated with antibiotics for 7 days demonstrates that the metabolite at *m/z* 160.1 is decreased in the colon of antibiotic-treated mice compared to controls. An image of carnitine (*m/z* 162.1) is shown as a control to demonstrate that metabolites are not universally decreased in the antibiotic treated mice (a). A box plot of *m/z* 160.1 ion intensity (normalized by TIC) in antibiotic treated mice colons compared to controls (b). Statistical analysis was performed by t test (*, $P \leq 0.05$) n=4. Bars represent mean +/- standard deviation. 16S rRNA gene sequencing and analysis for the gut microbiome was carried out on faeces from one week ABX treated mice and compared to untreated controls. (c) Rarefraction analysis of 16s rRNA gene sequences between ABX and No ABX samples. (d) Principal Coordinates Analysis (PCoA) of unweighted UniFrac distances of 16S rRNA genes. (e) Taxaplots depicting the relative abundance of phyla within the gut microbiome with and without ABX treatment. ABX treatment is causing a small number of more dominant operational taxonomic units (OTUs) to become depleted allowing the establishment of unique OTUs which subsequently increase their proportion within the population.

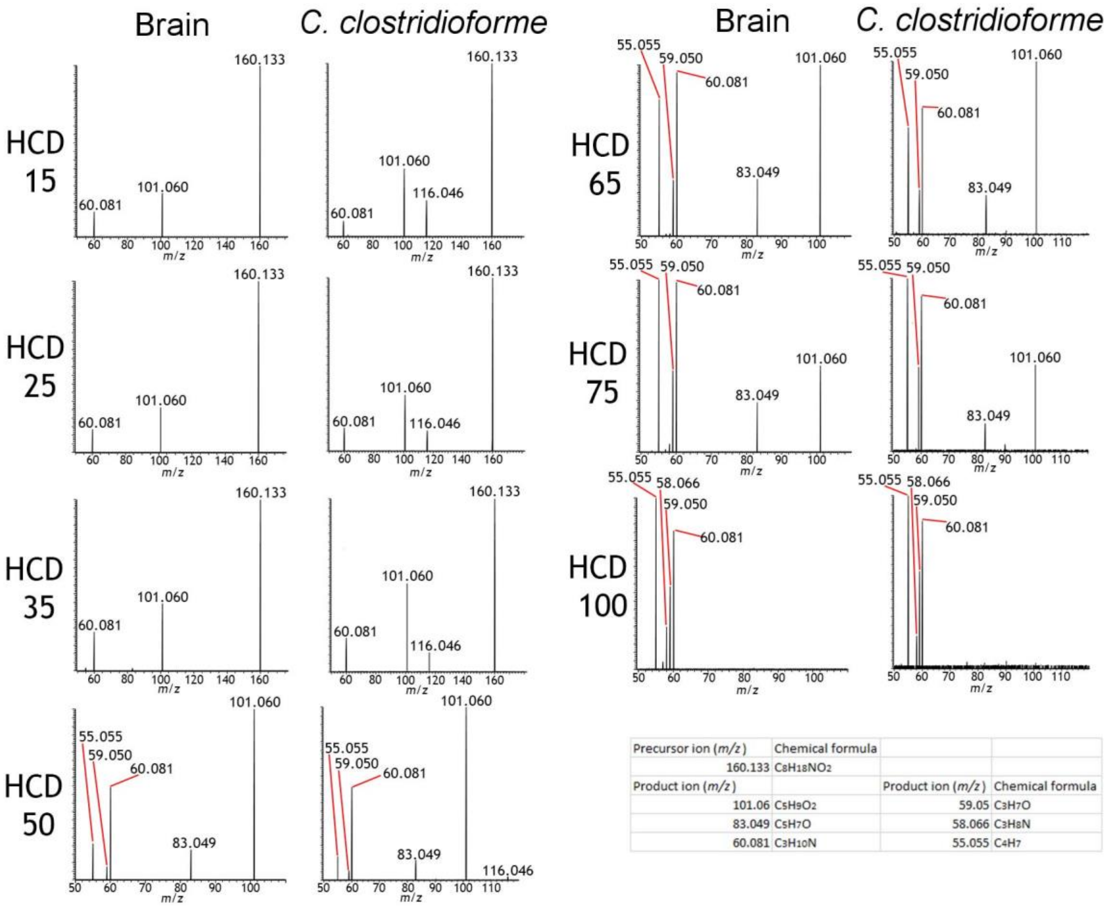

Precursor ion (m/z)	Chemical formula		
160.133	$C_8H_{18}NO_2$		
Product ion (m/z)		Product ion (m/z)	Chemical formula
101.06	$C_5H_9O_2$	59.05	C_3H_7O
83.049	C_5H_7O	58.066	C_3H_8N
60.081	$C_3H_{10}N$	55.055	C_4H_7

Fig. S4. MS/MS analysis of the metabolite found in the brain and the metabolite of the same *m/z* (160.133) produced by *C. clostridioforme* was undertaken. The corresponding smaller product ions produced across a range of HCDs indicated that the metabolites are the same. The peaks at *m/z* 60.081 and 58.066 indicate the presence of a trimethylamine group. The difference between product ions at *m/z* 101.060 and 55.055 indicates the loss of a carboxyl group, also consistent with the proposed structure.

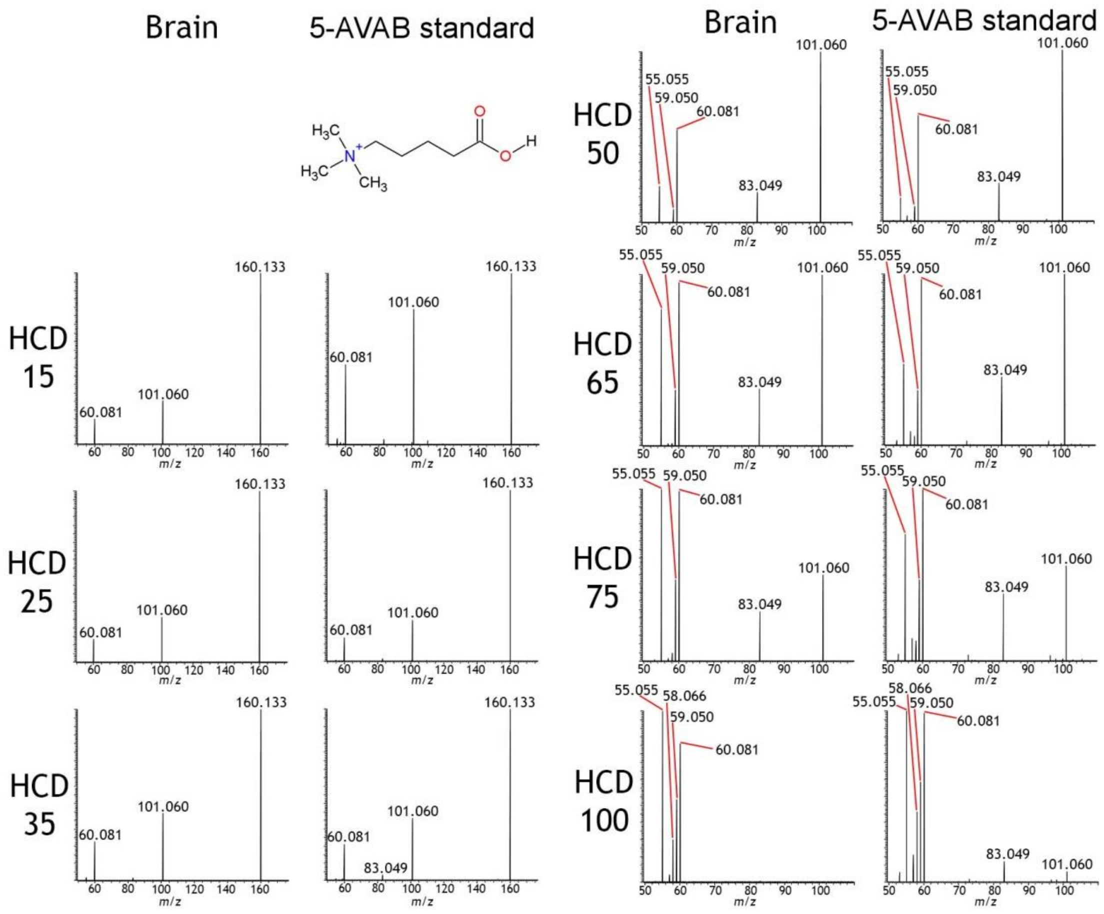

Fig. S5. Mass spectra from the MS/MS analysis of the synthesized standard 5-AVAB compared to the endogenous metabolite in the brain at *m/z* 160.133. MS/MS analysis was performed at various energies, HCD15, 25, 35, 50, 65, 75 and 100. The chemical structure of the synthesised standard is shown. At the lower collision energies (HCD15-35) a full spectrum is shown, *m/z* 50-180, but at the higher energies (HCD50-100) the spectrum is zoomed to show a range of *m/z* 50-120. At HCD 65 and 75 a distinct difference in the peak at 55.055 is noted, with it significantly lower in 5-AVAB compared to *m/z* 160.133 from the brain.

Fig. S6

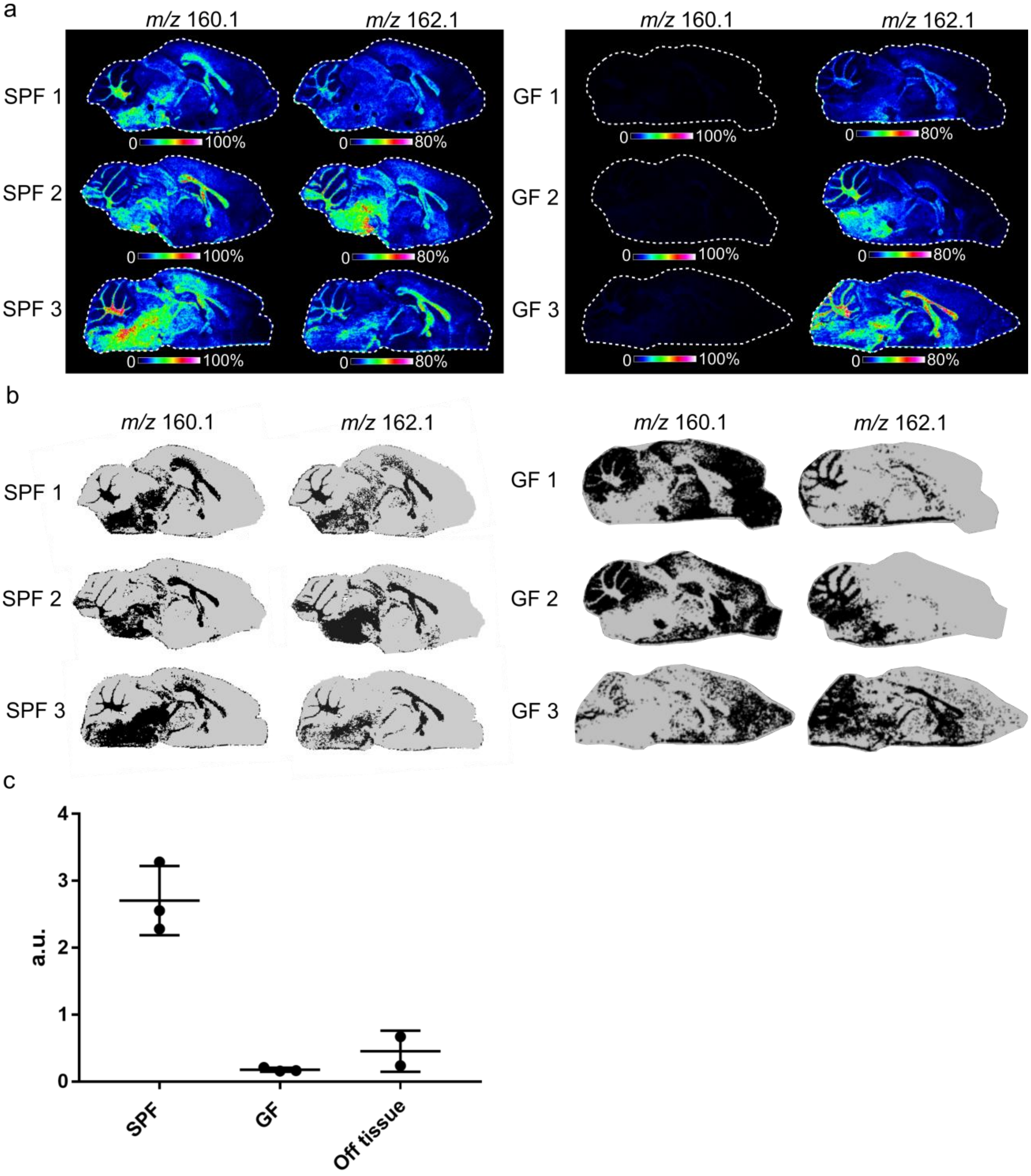

Fig. S6. Conversion of MALDI-MSI from intensity heatmaps (a) into binary images (b) showing the high (black) and low (grey) intensity regions of the ion images of *m/z* 160.1 (3M-4-TMAB/4-

TMAP) and *m/z* 162.1 (carnitine) in GF and SPF mouse brains (b). The binary image for each tissue section was generated by thresholding the ion intensities of that individual section for each *m/z* of interest, rather than the whole panel of images. Therefore in the case of *m/z* 160.1, in the GF mice while there is little to no signal observed (a), the minimal signal can be seen to localize to different anatomy than the *m/z* 162.1 (carnitine)(b). The overlap of these regions was then calculated as the percentage of pixels in the high intensity region of the *m/z* 160.1 image that were also high intensity in the *m/z* 162.1 image. Additionally, Pearson's correlation co-efficient between the two images was calculated for each tissue across three biological replicates (SPF1, SPF2 and SPF3, and GF 1, GF2 and G3); with a coefficient of 1 indicating perfect co-localization and -1 indicating no co-localization whatsoever (SPF1 overlap 92.85714, Pearson coefficient 0.921547; SPF2 93.20652, 0.906855; SPF3 63.20542, 0.609946), (GF 1 overlap 44.51, Pearson coefficient -0.12976; GF 2 58.31, -0.00027; GF 3 53.38, 0.177441). The binary images of *m/z* 160.1 do not reflect the difference in abundance of signal in GF and SPF brains. Differential thresholding was necessary to enable co-localization analysis of m/z 160.1 and carnitine (*m/z* 162.1) in GF brain due to the fact that the signal detected in the GF brain for *m/z* 160.1 was negligible (i.e, less than that of the off target control) as depicted in (c). All mice from all conditions were thresholded separately to account for potential variances in intensities between tissue sections related to minor differences in concentrations between animals. A graph of abundance of signal at *m/z* 160.1 is shown to compare levels in the corpus callosum of the SPF brain (area of high abundance of *m/z* 160.1), the corpus callosum of the GF brain and an off tissue negative control area (c). The graphs shows the level of signal detected in GF brains is similar to levels off-tissue, therefore the binary image generated for GF brains is likely generated from random background signal.

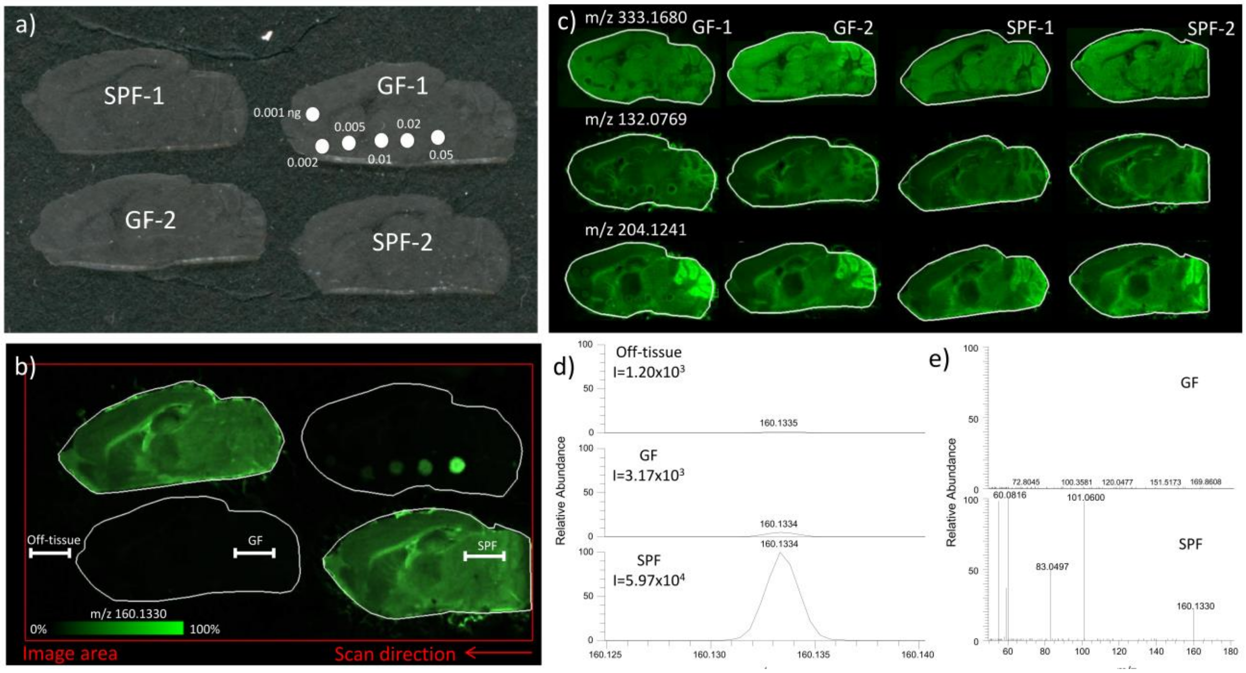

Fig. S7. Additional information to fig. S5. (a) Optical scan of slide setup used for quantification experiments. (b) DESI-MSI image shown in Fig. 5 as recorded. Red box indicates the whole image area data was acquired over. Bar inserts indicate where data was exported to generate panel (d). (c) Images of three endogenous molecules that show unaltered distribution over all four tissues analysed. (d) Zoom into mass spectrum at *m/z* 160.0-160.20 showing peaks at bar inserts in panel (b). (e) MS/MS data acquired over GF and SPF mouse brains.

To avoid any batch effect occurring in our MSI analysis, all tissues are sectioned and analysed in a randomised fashion. In DESI-MSI, images are acquired as horizontal line scans, in each line acquiring signal for GF, SPF and off-tissue areas. All images shown are unnormalized to show measurement stability. Images of endogenous molecules shown in panel (c) show that change in *m/z* 160.133 was not due to any systematic effect occurring during sample preparation or MS analysis. Data in panel (d) shows that signal at *m/z* 160.133 are detected off-tissue areas and both on SPF mice as well as on GF, signal is 18-fold increased in SPF mice over background signal. Levels in GF and negative control regions are of comparable background abundance. MS/MS

analysis was performed on SPF and GF brains to investigate the structural relationship between signal observed in both tissues (e). Signal from SPF brain is consistent with data recorded for 3M-4-TMAB/4-TMAP. No MS/MS spectrum could be obtained on GF brain. This suggests that low level signal observed in background and GF mice is an isobaric compound of different chemical nature. This is further suggested by comparison of data shown in Figure 4 and Figure S7. Spatial distribution of signal at *m/z* 160 in GF mice is different from that seen in SPF mice, suggesting compounds of different chemical nature and function. Furthermore, the identification of the metabolites was performed by NMR and the structure obtained is consistent with results from MS/MS analysis from brain tissue. Figure S1(e) shows that prominent fragments for 3M-4-TMAB/4-TMAP are not found in GF brains and off-tissue, consistent with the absence of these metabolites from GF tissues.

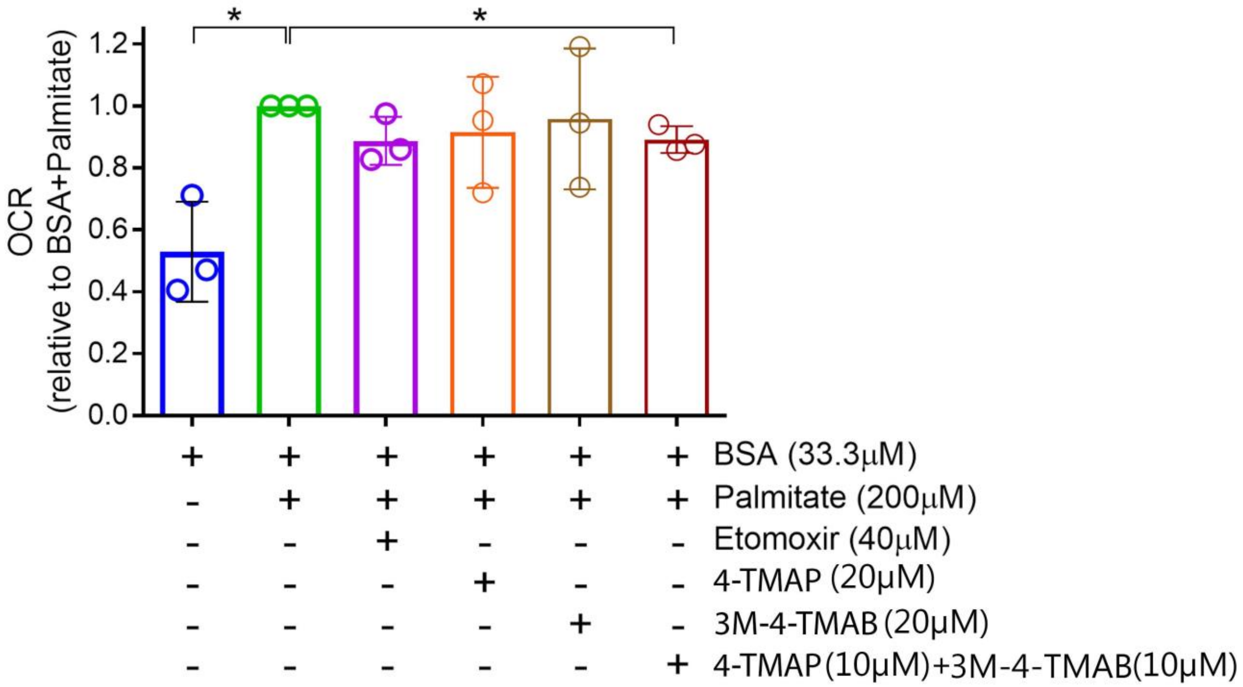

Fig. S8. OCR was used as an indicator of FAO in the presence of 3M-4-TMAB and 4-TMAP at the indicated concentrations and in the absence of carnitine supplementation. Oxidation of palmitate is significantly reduced in primary murine myelinating CNS white matter cultures in the presence of a combination of 3M-4-TMAB and 4-TMAP. Etomoxir an irreversible inhibitor of FAO, was used as a control. The OCR values are relative to the BSA+ palmitate condition. Each symbol represents the relative mean value for one experiment (n=3 independent experiments). The neural precursors used to generate the model of CNS white matter were obtained by pooling dissociated spinal cord cells from all embryos from a single pregnant female mouse. A one sample t test was used to test the statistical significance against the relative value 1. *P* values ≤ 0.05 were considered significant. **p*<0.05, and **** *p*<0.0001. Bars represent mean +/- s.d.

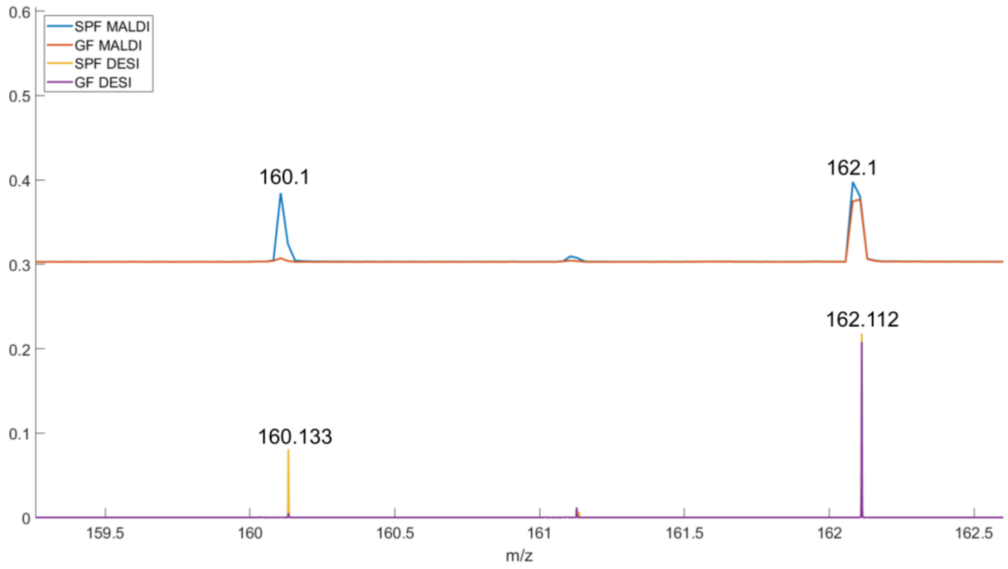

Fig. S9. Average mass spectra from MALDI-MSI and DESI-MSI results showing peaks at *m/z* 160.133 (3M-4-TMAB/4-TMAP) and *m/z* 162.112 (carnitine) from GF and SPF mouse brains.

Table S1. Summary of the top correlations with Pearson's coefficient > 0.5 for *m/z* 160.1 (3M-4-TMAB/4-TMAP) for the SPF brain tissue sections shown in Fig. 4.

m/z	SPF 1	SPF 2	SPF 3
204.1609	0.812962	0.725836	0.723954
258.167	0.682728	0.589967	0.630207
**162.0812**	**0.646327**	**0.641919**	**0.625101**
296.1213	0.564043	0.575823	0.562091
175.1077	0.624785	0.595438	0.545444
348.1271	0.570441	0.40996	0.545431
307.0975	0.459149	0.535216	0.529339
825.8807	0.489185	0.485021	0.524767
847.8832	0.509648	0.446728	0.518076
849.8834	0.515639	0.443699	0.51637

Summary of the top correlations with a Pearson's coefficient > 0.5 for *m/z* 160.1 (3M-4-TMAB/4-TMAP) for the SPF brain tissue sections shown in Figure 4. The third highest correlation is for carnitine *m/z* 162.1 (indicated in bold) for all three SPF brain tissue sections. The results show three biological replicates.
